# On the path to reclaiming Indigenous midwifery: Co‐creating the Maternal Infant Support Worker pilot program

**DOI:** 10.1002/ijgo.13918

**Published:** 2021-09-22

**Authors:** Naana Afua Jumah, Leanne Tyler, Roxanne Turuba, Lisa Bishop, Mary Tait, Anne Renaud, Christopher Mushquash

**Affiliations:** ^1^ Northern Ontario School of Medicine Thunder Bay ON Canada; ^2^ Centre for Rural and Northern Health Research Lakehead University Thunder Bay ON Canada; ^3^ School of Access and Success Confederation College Thunder Bay ON Canada; ^4^ Sioux Lookout Area Aboriginal Management Board Sioux Lookout ON Canada

**Keywords:** distance education, forced evacuation for birth, Indigenous midwifery, indigenous research methodology, perinatal care, rural and remote health, sweetgrass story weaving

## Abstract

**Objective:**

The aim of the Maternal Infant Support Worker (MiSW) pilot program was to implement a virtual training program for lay maternal–infant health providers in remote First Nations communities in Northwestern Ontario, Canada.

**Methods:**

The MiSW pilot program was administered jointly by a community college and a university and consisted of a 20‐week virtual course followed by a 9‐month mentored work placement in the community.

**Results:**

The MiSW pilot program was delivered successfully; 11 of 13 participants received a certificate from a community college. MiSWs provided culturally and linguistically appropriate care to women, infants, and families in their respective communities. MiSWs provided doula support in their communities—a first for our region since the policy of forced evacuation for birth was implemented. MiSWs developed a community of practice for ongoing education, as well as to support each other in their work.

**Conclusion:**

The MiSW pilot program demonstrated that it is possible to provide a virtual training program and then provide continued virtual mentorship as the participants work in their First Nations communities. By prioritizing Indigenous voices above those of the research team, we were able to gain the trust of the MiSWs and maintain engagement with communities.

## Synopsis

The Maternal Infant Support Worker pilot program successfully implemented a virtual training program for lay maternal–infant workers living in remote communities.

## 1 INTRODUCTION

Until the 1960s, many Indigenous women living in Northwestern Ontario, Canada gave birth in their home communities. These were moments of celebration and provided the mother and the newborn with invaluable community support.^1^ After this time, a policy of evacuation for birth was implemented, forcing women to deliver away from their family and community.^2^ Only six towns in the region provide maternity services and most care providers are non‐Indigenous. As a result, women living in remote First Nations communities have to travel in order to give birth, either by air from the 49 fly‐in Indigenous communities or by ground transportation for the remaining road and rail access communities. Women evacuated for birth experience loneliness and disconnection during the 2–12 weeks they are away from their families and communities.^3^

To exacerbate matters, Indigenous communities in the region began experiencing an epidemic of opioid misuse approximately 15 years ago.^4^ Although community treatment for opioid misuse has improved access to care,^5^ the 28% of First Nations women in remote and rural communities who use opioids during pregnancy continue to face a number of challenges,^6^ including delays returning home while their infant receives care for neonatal abstinence syndrome and fragmented care as communication between the hospital and the community nursing station is poor.^7^

The Maternal Infant Support Worker (MiSW) pilot program was a community‐based intervention that aimed to increase the health system's capacity to provide integrated maternity, mental health, and addiction care to pregnant and parenting women affected by opioids, living in rural and remote First Nations communities in Northwestern Ontario, Canada. This article describes the implementation of the MiSW pilot program and the co‐creation of the pilot program evaluation.

## 2 METHODS

### 2.1 Ethical space

The MiSW pilot program was carried out using the OCAP^®^ ^8^ and the Tri‐Council Policy Statement 2^9^ principles, which govern research involving Indigenous peoples in Canada. This program also used the Truth and Reconciliation Conciliation Commission of Canada Calls to Action^10^ and the Canadian Inquiry into Missing and Murdered Indigenous Women and Girls Calls to Justice^11^ as a guiding framework (Table 1). The MiSW pilot project training and mentored practice phases were approved by the research ethics boards at Confederation College and Lakehead University.

**TABLE 1:** Ethical framework for the Maternal Infant Support Worker pilot program

Organization	Document
First Nations Information Governance Centre	OCAP^®^
Government of Canada	Tri‐council Policy Statement 2, Chapter 9
Truth and Reconciliation Commission of Canada	Calls to Action 18, 22, and 23
Canadian Inquiry into Missing and Murdered Indigenous Women and Girls Calls to Justice	Calls to Justice 3.1 and 7.1

### 2.2 Organizing framework

Indigenous grassroots theory and the sweetgrass story weaving methodology described by Wabie,^12^ an Algonquin researcher from Northern Ontario, formed the foundation for creating and evaluating the MiSW pilot program. This methodology centers Indigenous voices and focuses on the interconnectedness of mind, body, and spirit. The symbolism of sweetgrass was used as the organizing framework: surveying sweetgrass—preparation for the MiSW pilot program; gathering sweetgrass—MiSWs gathering information through their training; braiding sweetgrass—MiSWs consolidating their knowledge and skills through mentored practice; talking sweetgrass stories—community‐level impact of the MiSW pilot program; and gifting sweetgrass—future goals of the MiSWs. This article encompasses the areas of surveying, gathering, and braiding sweetgrass.

### 2.3 Collaborating organizations

The MiSW pilot program was developed in 2013 by First Nations Elders, the Sioux Lookout Area Aboriginal Management Board (SLAAMB), the Sioux Lookout Meno Ya Win Health Centre, and Confederation College (see https://www.youtube.com/watch?v=bK_bzr2aJqs). At that time, funding was only secured for curriculum development. Funding to implement the MiSW pilot program was obtained in 2018, with additional collaboration from the Northern Ontario School of Medicine and Lakehead University (Table 2).

**TABLE 2:** Collaborating organizations

Sector	Organization
Academic stakeholders	Confederation College
	Lakehead University Centre for Rural and Northern Health Research
	Northern Ontario School of Medicine
Government and Indigenous stakeholders	Sioux Lookout Area Aboriginal Management Board
	Ontario Ministry of Health and Longterm Care
Hospitals	Sioux Lookout Meno Ya Win Health Centre
	Thunder Bay Regional Health Sciences Centre

### 2.4 Program description

The MiSW pilot program was a facilitated online training program for lay maternal–infant health providers consisting of two phases: training and mentored practice. The training phase consisted of 18 weeks of facilitated online coursework shouldered by 2 weeks of in‐person sessions, including a hospital placement in a prenatal clinic and on labor and delivery. The mentored practice phase consisted of 9 months of working in the community with the support of the course instructor and research team. Each participant received a tablet computer with pre‐loaded curriculum that was also used to connect to virtual sessions. Participants were required to submit weekly self‐reflections throughout the pilot.

### 2.5 Program administration

The training phase was administered by Confederation College and the mentored practice phase was administered by Lakehead University. The course instructor submitted weekly notes on participation, pertinent themes, comments, and concerns from the participants, as well as documenting activities, programs, or training that the participants were involved with in their communities. This information was reviewed weekly by the research team and used to refine the curriculum. Advice and guidance were sought from health directors as issues relevant to the program or their respective community arose.

### 2.6 Inclusion criteria

Health directors from SLAAMB communities were invited to participate by the research team. Health directors then nominated a community member who was actively working in maternal–infant health and who met the following inclusion criteria: minimum of Grade 10 and/or assessment of lived experience by the selection committee; recommendation by the community; interview by the research team; age 18 years or older; comfortable with technology; and ability to commit 20 h per week to the pilot program. Nominees then provided their written consent to participate in the MiSW pilot program.

### 2.7 Surveying sweetgrass—preparation for the MiSW pilot program

The first meeting for the MiSW pilot project was held on June 15, 2018. During this meeting, a course instructor was identified who had nursing experience and who had the respect of the First Nations communities in the region. The roles and responsibilities of each of the collaborating organizations were defined and a timeline for the pilot program was established (Table 3).

**TABLE 3:** Maternal Infant Support Worker course schedule

Cohort	Dates	Location of training
First cohort	November 5–18, 2018	Sioux Lookout
	November 19 to December 21, 2018	Home community
	December 24, 2018 to January 4, 2019	Christmas break—no classes
	January 7 to March 29, 2019	Home community
	April 1–5, 2019	Sioux Lookout Meno Ya Win Health Centre
	April 8 to December 31, 2019	Implementation phase in home community
Second cohort	April 6–12, 2019	Sioux Lookout
	April 15 to August 16, 2019	Home community
	August 18–23, 2019	Sioux Lookout Meno Ya Win Health Centre
	August 26 to May 29, 2020	Implementation phase in home community

A review of the course objectives, which were structured across five dimensions (health system navigator, communicator, expert, health promoter, and professional) was undertaken (Table 4). The first course objective was to acknowledge that funding for maternal–infant workers may come from a single source or from multiple sources, and, as a result, the reporting structure, roles, responsibilities, and the work environment for each participant may be different. As a standardized job description does not exist, our goal was to support the participants in their role as defined by the community.

**TABLE 4:** Maternal Infant Support Worker pilot program objectives

Dimension	Course objectives
Health system navigator	Act within the role of the Maternal‐infant Support Worker by following established policies and procedures, service plans in both institutional, and community‐based settings Apply a working knowledge of the healthcare system/function as a navigator through the system
Communicator	Develop ability to work and communicate as part of an inter‐professional team Communicate effectively with First Nations clients/families and others in the healthcare system Communicate accurately both verbally and in writing using applicable terminology in the workplace
Expert	Apply general knowledge of anatomy and physiology, stages of pregnancy, medical process and procedures involved in giving birth, nutrition, health, and wellness
Health promoter	Develop strategies to promote positive choices for healthy pregnancies, effective maternal support in communities Promote understanding and participation in health care by women and their families
Professional	Demonstrate a professional attitude and behavior appropriate to the healthcare setting

Prior to the initiation of the MiSW pilot program, the research team had informal conversations with health directors and administrators in the SLAAMB communities. Three health directors were either themselves Indigenous midwives until the policy of forced evacuation for birth ended Indigenous midwifery in the region or had Indigenous midwives in their families. There was an overwhelming positive response for this type of program. Only one health director provided words of caution, that, although this type of program is a step in the right direction, it is not a replacement for traditional knowledge systems around pregnancy and infant care, traditional skills around birth, and traditional ways of sharing knowledge.

Since the initiation of the MiSW pilot program in 2013, Canada's relationship with Indigenous peoples has been evolving, guided by the report of the Truth and Reconciliation Conciliation Commission. As a result, a review of the curriculum was undertaken. Of the 40 videos that were originally created for the program, only five contained Indigenous content. Curriculum items that were no longer deemed appropriate were removed and replaced by other resources, including the addition of more Anishnaabe‐ and First Nations‐based content (Table 5), at the discretion of the course instructor and research team. Table 6 contains a summary of the weekly virtual curriculum content.

**TABLE 5:** Indigenous‐led workshops and Indigenous course content included in the Maternal Infant Support Worker pilot program

Organization	Workshop
Zaagi'idawin	Indigenous Doula Training
Mental Health Commission of Canada	Mental Health First Aid First Nations
Janet Fox	Traditional Parenting Training

Organization	Course Content
National Aboriginal Council of Midwives	Stories and teachings about pregnancy, birth, and infant care
Health Nexus/Best Start	Supporting the Sacred Journey; from Preconception to Parenting for First Nations Families in Ontario Why Am I Poor? First Nations Child Poverty in Ontario Prescription Drug Misuse in Pregnancy & Parenting, A Report for Service Providers Working with First Nation Women in Ontario A Sense of Belonging: Supporting Healthy Child Development in Aboriginal Families
Sioux Lookout Meno Ya Win Health Centre	Healthy Choices for Healthy Babies Resources
Early Childhood Development and Intercultural Partnerships	Beginning the Journey of Fatherhood, A guide for Aboriginal Men; Jessica Ball 2012
British Columbia Provincial Health Services Authority	Our Sacred Journey: Aboriginal Pregnancy Passport

**TABLE 6:** Maternal Infant Support Worker pilot program virtual curriculum content summary

Week	Course content
1	Anatomy
2	Physiology
3	Healthy pregnancy—first trimester
4	Substance use in pregnancy
5	Healthy pregnancy—second trimester
6	Healthy pregnancy—third trimester and the placenta
7	Labor and delivery
8	Healthy pregnancy—post‐partum the first 6 seeks
9	Dad's health and role in pregnancy
10	Hypertension in pregnancy
11	Diabetes in pregnancy
12	Complications in pregnancy/participant topics
13	Professional development: confidentiality, privacy, documentation, duty to report
14	Mental health
15	Breastfeeding
16	Newborn care
17	Infant growth and development
18	Healthy sleep

## 3 RESULTS

### 3.1 Gathering sweetgrass—the MiSW training phase

**FIGURE 1:**
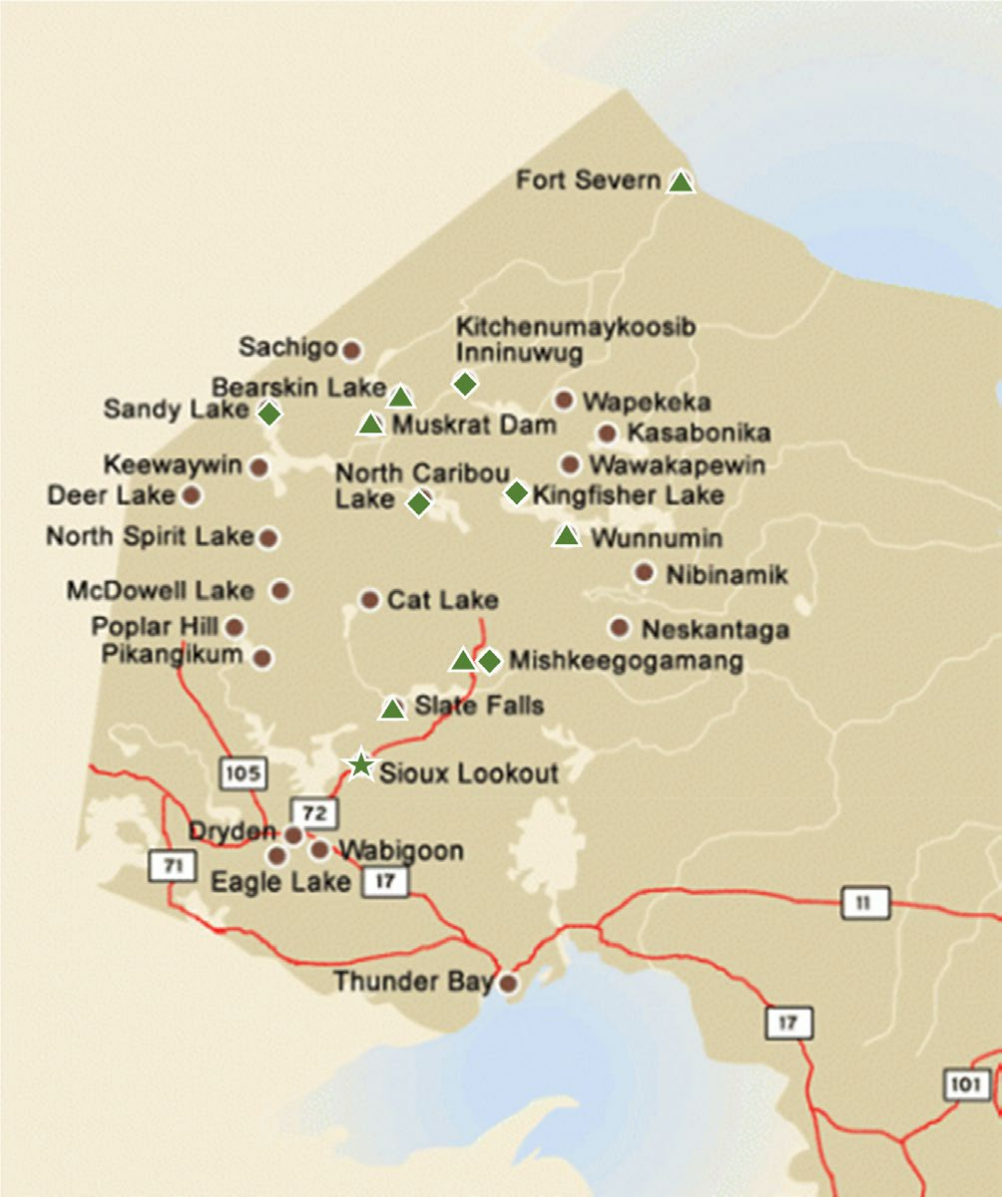
Map of Northwestern Ontario, Canada. Sioux Lookout Area Aboriginal Management Board communities participating in the Maternal Infant Support Worker pilot program

Two cohorts of six individuals participated in the pilot program (Figure 1, Table 7). As the first cohort progressed through the virtual training, it was evident that the video‐based meetings were not always possible due to the availability of the internet and internet bandwidth limitations in remote communities. Telephone communication was the backup when the internet failed.

**TABLE 7:** Maternal Infant Support Worker pilot program participant summary

First cohort	Total participants enrolled, *n* = 7 1 participant withdrew from program after week 8 1 participant added to program during week 9 6 participants completed training phase	
Second cohort	Total participants enrolled, *n* = 6 1 participant withdrew from program after week 13 5 participants completed training phase	
First Nations communities	Bearskin Lake, *n* = 1 Kingfisher Lake, *n* = 2 Mishkeegogamang, *n* = 3 North Caribou Lake, *n* = 1 Slate Falls, *n* = 1	Fort Severn, *n* = 1 Kitchenumaykoosib Inninuwug, *n* = 1 Muskrat Dam, *n* = 1 Sandy Lake, *n* = 1 Wunnumin Lake, *n* = 1

Although the inclusion criteria contained educational attainment, we did not collect data on this because the intent of the pilot program was to validate the skills and knowledge that the individuals had as recognized by their community. Through discussions with the participants, it was clear that most participants had not completed high school and, therefore, they had been nominated by their communities based on lived experience.

Nine of the 13 participants spoke Ojibway or Ojicree as their first language. Through observation we identified a few participants who probably had low literacy in English. This issue was never addressed explicitly. Rather, we adjusted the presentation of materials to meet their learning needs. The other participants also began translating for their colleagues or providing their interpretation of the written information, which stimulated discussion and allowed for inclusion of everyone in the cohort. Weekly journaling was a part of reflective practice in the curriculum. However, when literacy concerns were identified, this requirement was replaced by weekly individual communication with the course instructor.

Additional factors competing for the attention of participants during the pilot were the demands of their jobs and personal or community emergencies. The course instructor was available for individual tutorials when needed, regardless of the reason. Although there was a decline in participation in the virtual group meetings as the cohorts progressed, the course instructor had contact with all participants individually on a weekly basis, except in circumstances of illness or emergency.

In terms of overall participation, one participant in the first cohort withdrew from the pilot program for personal reasons. The nominating community put forward a replacement. After discussions with community representatives, it was determined that an accelerated catch‐up program could be offered and the request was accommodated. One participant in the second cohort missed the initial in‐person gathering and then did not participate in group or individual meetings. Efforts to increase engagement were unsuccessful. At the 13‐week mark, the participant was too far behind to successfully complete the pilot program. After discussions, the participant withdrew from the pilot program.

The remainder of the participants graduated from the pilot program and were gifted a braid of sweetgrass upon graduation. The research team underestimated the significance that graduation held for the participants, their families, and the communities. The ceremonies received widespread attention in local and regional media, as well as thousands of impressions on social media, including tweets from the Minister of Indigenous Services Canada.

During the final in‐person week with the first cohort, one half‐day was dedicated to developing evaluation materials. For the mentored practice phase, the participants recommended continuing reflective practice using oral documentation. Weekly visit logs and touchpoint logs that were largely checkbox based were also developed and approved by the participants. For the overall pilot evaluation, the participants recommended interview‐based tools to facilitate data collection. Key informant interview guides were developed for the course instructor, community administrators, mothers, and MiSWs.

### 3.2 Braiding sweetgrass—MiSW mentored practice phase

Following graduation, the MiSWs continued to work in their respective communities and received mentorship for 9 months in the form of weekly virtual meetings that had two aims: developing case management skills and addressing the specific learning needs of the participant. Table 8 summarizes the topics that were generated by the participants. This phase of the pilot program was completed in full by the first cohort and only partially by the second cohort due to interruption from the COVID‐19 pandemic.

**TABLE 8:** Maternal Infant Support Worker pilot program mentored practice participant driven content summary

Dimension	Course content
Health system navigator	Registering newborns who have been adopted through the traditional process Travel for labor and delivery during the pandemic Referrals to community‐based supports
Communicator	Advocating for patients at the nursing station Communication within the circle of care Making presentations and giving interviews
Expert	Urinary tract infections in pregnancy Natural methods of inducing labor at term safely Round ligament pain Breastfeeding, latch issues, mastitis Early labor Upper respiratory tract infections Eating disorders in pregnancy Miscarriages
Health promoter	Support for in‐community deliveries Techniques for delivery programming at the community level
Professional	Training opportunities for professional development Sharing of online, Indigenous‐based knowledge tools Collegial support for accomplishments

Among the many activities during the mentored practice phase (Table 9), the MiSWs also provided doula support. Two MiSWs supported women during births at their respective nursing stations. MiSWs also provided doula support to women who presented to the nursing station in labor prior to evacuation to a maternity hospital. One urban MiSW provided doula support to Indigenous women in hospital until the COVID‐19 pandemic, at which time she transitioned her role to coordinating virtual prenatal care appointments for Indigenous women living in remote communities.

**TABLE 9:** Maternal Infant Support Worker activities during the 9‐month mentored practice

Dimension	Activities
Health system navigator	Participating in a pilot to provide prenatal education in community and facilitate prenatal care via OTN Consulting for the Registered Nurses of Association of Ontario on policies and guidelines for ethical work with Indigenous peoples Working with nurses, community health representatives, the health director, chief and council
Communicator	Providing services in Indigenous languages of the region—Ojibway, Ojicree, and Cree Providing linguistic and cultural interpretation Facilitating communication between clients, health professionals, and nursing stations Presenting to media and at health professional conferences and meetings
Expert	Seeking out additional training in breastfeeding Doula support for laboring women at two regional hospitals and in the community Conducting home visits with clients One on one visits with clients Facilitating group activities for families
Health promoter	Prenatal nutrition programming Healthy Baby Healthy Children programming Choose Life programming Community baby showers Education around postpartum depression Online support for pregnancy and infant loss Adolescent healthy lifestyle programming
Professional	Creating and engaging in an online community of practice to share resources, exchange best practices, and participate in professional development Establishing a peer support network to share successes and challenges

Below is an example that illustrates the role and impact of the MiSWs in the community that unfolded over a number of days, as told to the course instructor.

An MiSW in community A was contacted via a mobile messaging app by a woman in distress with a concealed pregnancy in community B. The MiSW sought advice from her colleagues while she supported the woman and diagnosed labor and ruptured membranes. The MiSW encouraged the woman to go to the nursing station, remained with her during labor and delivery at the nursing station. The woman and her baby were transferred by air to the hospital after delivery. The MiSW got back in touch with the woman at the hospital to provide ongoing support and again when she was discharged home to her community.

The MiSWs developed more confidence in themselves in the safe and collegial space created during the mentored practice phase. They began to rely less on the course instructor and more on each other for support and information. The MiSWs started an online “community of practice” for themselves that continues to this day as a network for support, information, and sharing of professional development opportunities.

## 4 DISCUSSION

The MiSW pilot program faced four major challenges. First, restructuring at Confederation College led to a personnel change that disrupted the relationship between the research team and the participants during the training phase for the first cohort. Second, following a change in government in 2018, the grant scheme funding the pilot project was terminated 1 week after the second cohort began the training phase. Funds were reallocated at SLAAMB and from other research grants, enabling the second cohort to continue.

Third, since the start of the COVID‐19 pandemic in 2020, all in‐person research was halted by Lakehead University, and all Indigenous communities restricted access to non‐community members. Despite this, the course instructor continued to mentor the MiSWs and help them translate their skills to new roles in the local pandemic response.

Finally, despite our efforts to co‐create evaluation materials for the mentored practice phase, the participants in both cohorts did not complete the logs or the weekly self‐reflections. The research team wondered if the logs represented an additional documentation burden, but a clear response to this question was not obtained. In keeping with Indigenous grass roots theory, the decision was made to abandon the log‐based evaluation in favor of centering the stories of the MiSWs themselves. They had ongoing dialogue with the course instructor regarding the type of cases they were seeing, as well as their reflections on their management of clients. The course instructor, with the consent of the participants in both cohorts, kept notes of the encounters.

## 5 CONCLUSION

The overall aim of the MiSW pilot program—to implement a virtual training program for lay maternal–infant health providers in remote First Nations communities and to support them through the first 9 months following graduation—was achieved. By prioritizing Indigenous voices above those of the research team, we were able to gain the trust of the MiSWs and maintain engagement with communities. Our next steps will be to complete the overall pilot program evaluation to assess the impact of the MiSWs in their communities.

## CONFLICTS OF INTEREST

The authors have no conflicts of interest.

## AUTHOR CONTRIBUTIONS

Under the lead of NJ, the authors LB, MT, AR, and CM contributed to the design of the study; LT and RT contributed to data collection and analysis. All authors contributed to manuscript writing and approved the final draft of the manuscript.
